# Myxoid liposarcoma of the leg: a case report

**DOI:** 10.1093/bjrcr/uaaf055

**Published:** 2025-11-18

**Authors:** Angelo Montana, Gabriele Mirabella, Giambattista Privitera, Stefano Bastoni, Antonina Parafioriti, Placido Romeo

**Affiliations:** Department of Imaging Diagnostics and Laboratory, Policlinico “G. Rodolico-San Marco”, Catania 95123, Italy; Department of Radiology, Umberto I Hospital, Enna 94100, Italy; Department of Imaging Diagnostics and Laboratory, Policlinico “G. Rodolico-San Marco”, Catania 95123, Italy; C.O.O., Azienda Socio Sanitaria Territoriale Gaetano Pini, Milan 20122, Italy; Pathology Department, ASST Pini-CTO, Milan 20122, Italy; Department of Imaging Diagnostics and Laboratory, Policlinico “G. Rodolico-San Marco”, Catania 95123, Italy

**Keywords:** MRI, mixoid liposarcoma, tumour of the soft tissue, radioterapy

## Abstract

Myxoid liposarcoma is the second most frequent subtype of liposarcoma, affecting predominantly the extremities, as seen in our case report. Diagnosing myxoid liposarcoma using imaging can be challenging due to its characteristics on both CT and MRI; for this reason, a combination of imaging techniques and histological patterns is necessary to accurately assess the tumour and subsequently make a preoperative planning. We present a case of myxoid liposarcoma arising from the intermuscular fascial planes of the anterior compartment of the leg.

## Introduction

Myxoid liposarcoma is the second most frequent liposarcoma subtype, accounting for 30%-40% of all liposarcomas. It predominantly affects the extremities and 80% of the tumours originate from the intermuscular fascial planes.^1^^,^^2^ This tumour subtype primarily affects young adults and typically contains a gelatinous myxoid matrix as histological pattern.

We present a case of myxoid liposarcoma arising from the intermuscular fascial planes of the anterior compartment of the leg. In order to better define the tumour boundaries and to guide the surgical intervention, we considered an integrated approach with MRI and ultrasound.

Myxoid liposarcoma can be difficult to diagnose because its imaging appearance is sometimes unusual. At MRI, it generally consists of a multilobulated mass with high water content that therefore appears hypointense on T1-weighted and hyperintense on T2-weighted sequences.^1^^,^^3^ These imaging characteristics may sometimes lead to confusion with cystic lesions. The presence of thin, unsharp fatty septa and diffuse enhancement after gadolinium administration, however, can be useful in the differential diagnosis. Such enhancement is also important for differentiating myxoid liposarcoma from benign cystic tumours and for planning the appropriate surgical approach.

The current case report demonstrates the rare presentation of a myxoid liposarcoma arising from the intermuscular fascial planes of the anterior compartment of the leg.

## Case report

A 50-year-old female presented in our department with a progressively enlarging mass in the anterior compartment of her right leg. This mass was noted from the patient approximately a few months ago as a small round mass that progressively increased its size. Initially the patient described the mass as painless and relatively soft upon palpation with no significant tenderness. There was no history of trauma and her medical history presented no issues. In the past months, during the size increasing, it became more painful. At the objective examination this mass was immobile, with irregular margins.

An ultrasound of the leg revealed a large hypoechoic mass, but its exact margins could not be defined.

**Figure 1.**
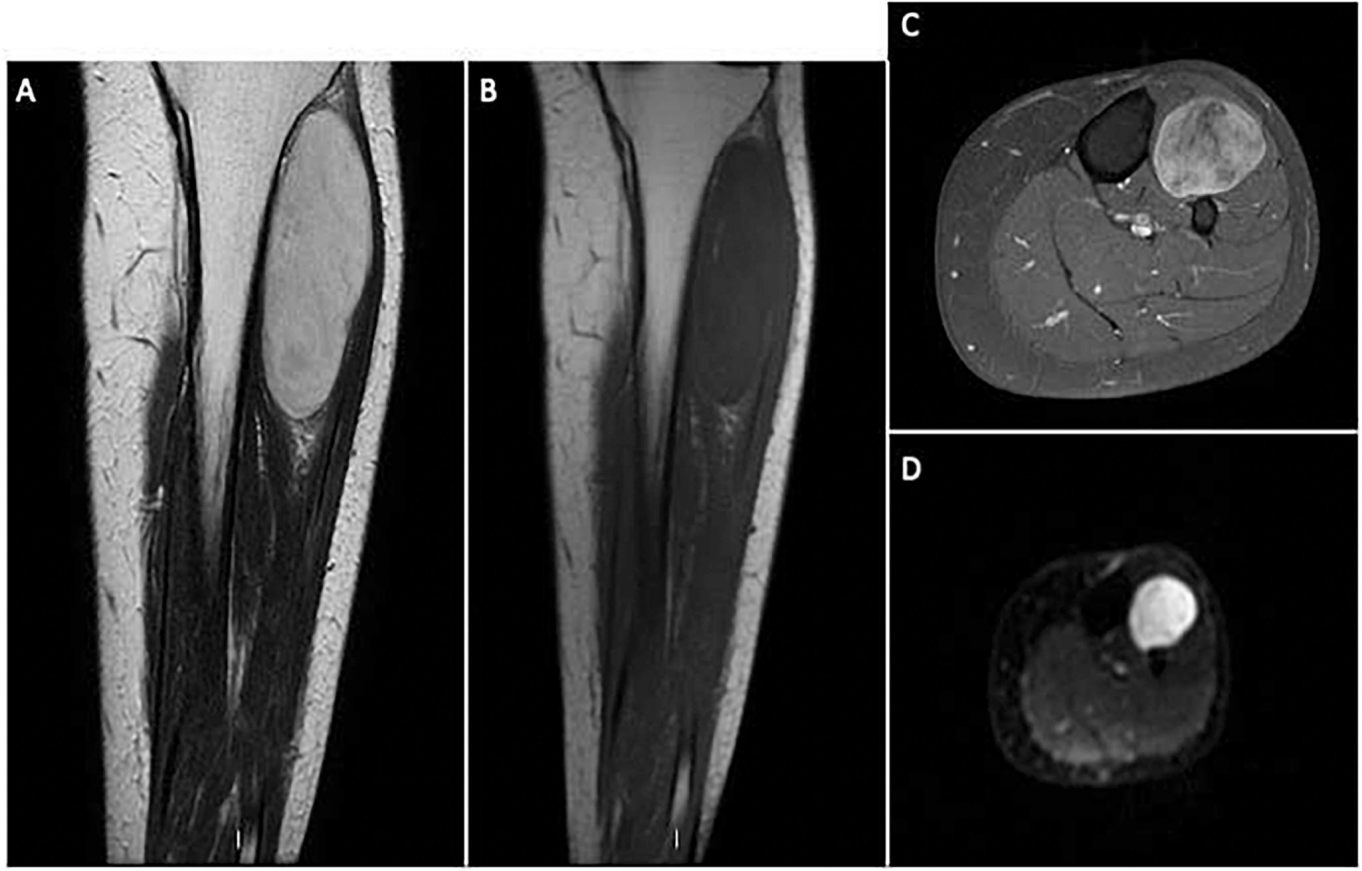
MRI of myxoid liposarcoma. (A) Coronal T2-weighted image; (B) Coronal T1-weighted image; (C) Axial T1 fat-saturated post-contrast image; (D) Diffusion-weighted image. The bulky solid mass shows oval morphology, a predominantly hyperintense signal on T2 with thin hypointense septa, a hypointense signal on T1, irregular diffusion restriction, and conspicuous post-contrast enhancement.

Due to the size and characteristics MRI were performed and revealed a large, heterogeneous mass within the intermuscular fascial planes of the anterior compartment of the leg with high signal intensity on T2 images and areas of low signal intensity due to the myxoid material. The imaging indicated minimal fat content, resulting in low T1 signal, with some hyperintensity spots, aligning with the diagnosis of myxoid liposarcoma. We also performed DWI and T1 Fat-Sat (FS) + contrast sequences showing respectively restriction and contrast uptake of the lesion (Figure 1).

**Figure 2.**
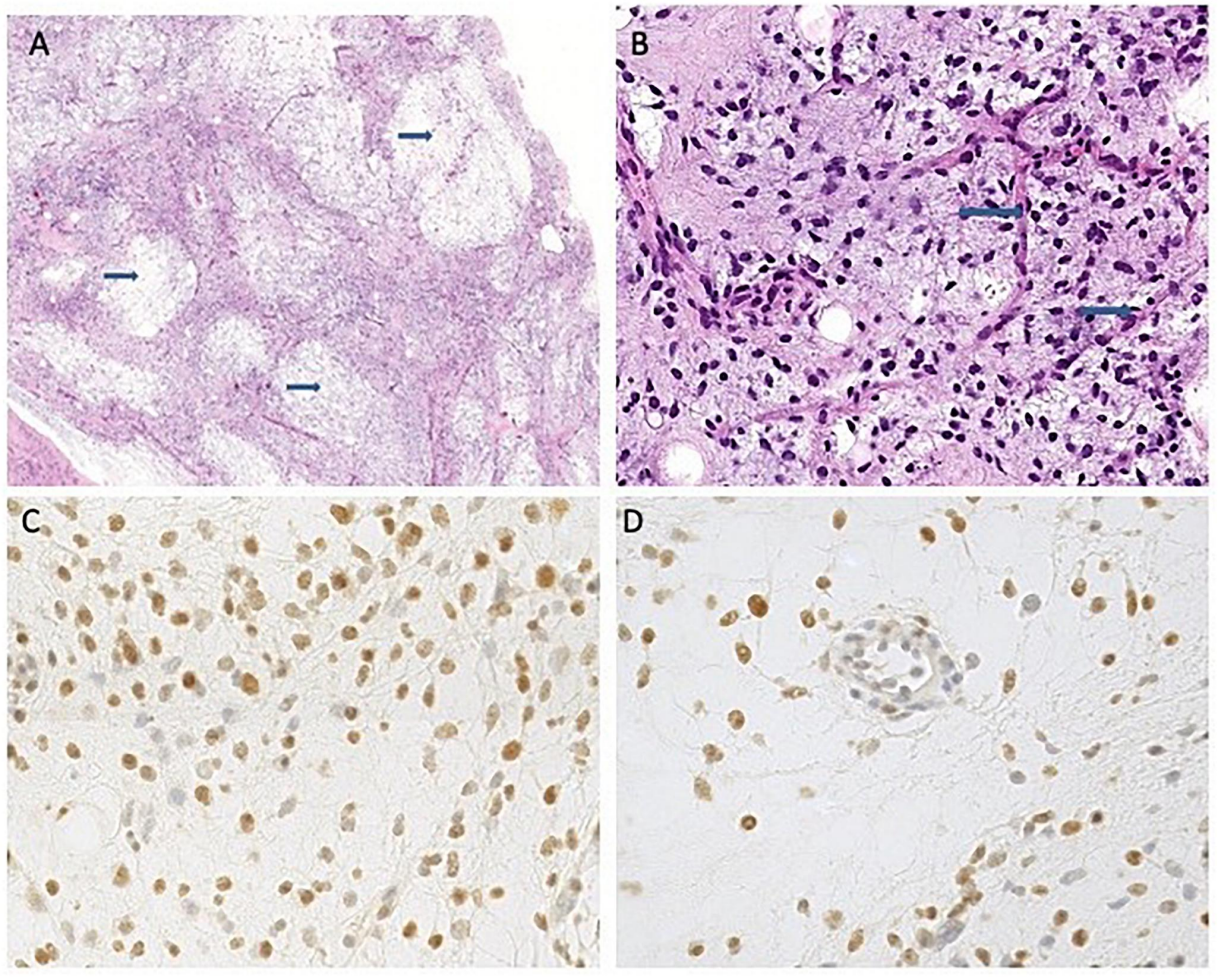
Biopsy confirmation of myxoid liposarcoma. (A) solid proliferation with myxoid stroma (arrows); (B) small round to oval cells and characteristic arciform vessels (arrows); (C, D) Immunohistochemistry for DDIT3 showing strong nuclear positivity.

A biopsy confirmed the presence of a myxoid pattern with a high cellularity (>15%); immunohistochemical staining were performed (Figure 2).

The pathology report demonstrated: DDIT3+, Retinoblastome+, S100 rare+; CD34-, MDM2-, CDK4-. Ki-67 5%.

**Figure 3.**
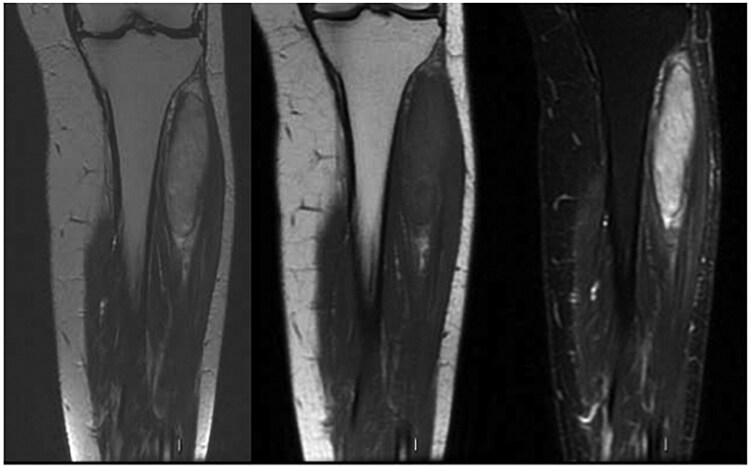
MRI after radiotherapy. (A) Coronal T2-weighted image; (B) Coronal T1-weighted image; (C) Coronal STIR image. Significant volumetric reduction of the liposarcomatous mass is evident.

After the results of the biopsy, due to the size of the lesion, was first performed radiotherapy and subsequently a new pre-operative MRI; (Figure 3) after that, the patient had a surgical resection.

**Figure 4.**
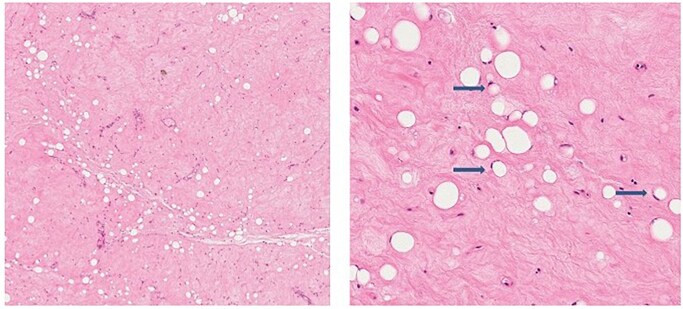
Histology after treatment. Hematoxylin and eosin staining shows extensive sclerohyalinosis, necrosis, and lipoblastic maturation (arrows).

Once the tumour was removed, it was sent to the pathology department for histology (Figure 4).

**Figure 5.**
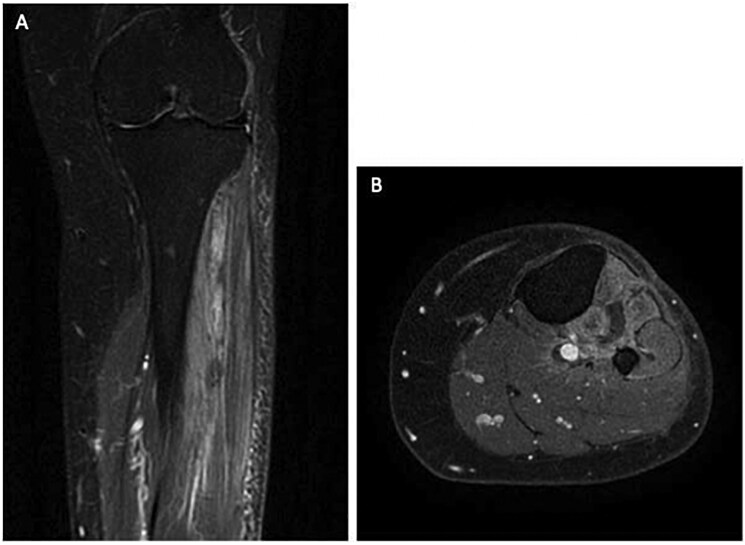
Post-surgical MRI of residual myxoid liposarcoma. (A) Coronal T1 fat-saturated post-contrast image; (B) Axial T1 fat-saturated post-contrast image. Minimal residual neoplastic tissue is visible as irregular contrast enhancement in the interfascial area.

Despite the aggressive treatment, the patient presented a residual lesion at the MRI we performed after 1 month from the resection (Figure 5). The residual prompted further discussion of follow-up and eventual treatment options, including additional surgery.

## Discussion

Myxoid liposarcoma is one of the most common subtypes of liposarcoma. Most patients diagnosed with this type of tumour are middle-aged, and it usually first presents as a painless, soft, slow-growing mass, as in our case.^1^

We presented a case of a tumour arising from the intermuscular fascial planes of the anterior compartment of tibial muscles, which is an uncommon site. This tumour could easily mimic a fluid-filled cyst on T2-weighted images due to its high signal intensity with a well-defined outline.^4^ In our case, MRI revealed a heterogeneous mass arising from the intermuscular fascial planes from the anterior tibial compartment with low-intensity T1 signal and high-intensity T2 signal.

Post-contrast imaging with gadolinium showed mild and heterogeneous enhancement, representing areas of increased cellularity and vascularity. These features are in concert with morphology from classic myxoid liposarcomas with scant fat and bright appearance on T2-weighted sequences. STIR sequence also was useful to differentiate myxoid liposarcomas from other soft tissue neoplasms like well-differentiated liposarcoma or lipoma.^4^

A major concern with myxoid liposarcoma is local recurrence and the ability to form distant metastases, both of which can occur in up to one-third of patients. Prognosis is largely influenced by the histological composition of the tumour itself, especially about the presence of a round cell component. Tumours containing more than 5% round cell component implicate poorer prognosis, and when the round cell component exceeds 25%, the tumour is defined as high grade.^5^ Surgical wide resection remains the standard treatment for myxoid liposarcoma, mainly for clear margins. For higher-grade tumours or those with significant round cell components, more aggressive resections may be involved, often involving the removal of surrounding muscle tissue as well. In this case, neoadjuvant radiotherapy to shrink tumour size in preparation for wide local excision was used.

## Conclusion

Liposarcomas are malignant mesenchymal tumours and the second most common soft tissue neoplasms; the myxoid is the second most frequent liposarcoma subtype.

This case of myxoid liposarcoma arising from the intermuscular fascial planes of the anterior compartment of the leg illustrates the difficulty in managing this aggressive tumour. It is essential for the radiologist to understand the differences between the subtypes of liposarcoma and their diverse anatomical presentations and imaging characteristics.

## Learning point

- myxoid liposarcoma can present in unusual sites, such as the muscular fascia of the anterior compartment of the leg
- the prognosis of the myxoid liposarcoma is significantly influenced by the percentage of round cells component
- DDIT3+ immunohistochemistry may support the diagnosis of myxoid liposarcoma
